# Supplementary material for: Dual function of HPF1 in the modulation of PARP1 and PARP2 activities
Source: Commun Biol. 2021 Nov 3;4:1259. doi: 10.1038/s42003-021-02780-0 (PMC8566583; doi:10.1038/s42003-021-02780-0)
Supplement: Supplementary file 2 — Supplementary Information [file 42003_2021_2780_MOESM2_ESM.pdf]

Supplementary material for

Dual function of HPF1 in the modulation of PARP1 and PARP2 activities

Tatyana A. Kurgina[1,2,3], Nina A. Moor[1,3], Mikhail M. Kutuzov[1], Konstantin N. Naumenko[1], Alexander A. Ukraintsev[1], Olga I. Lavrik[1,2]*

[1]Institute of Chemical Biology and Fundamental Medicine, SB RAS, Novosibirsk, Russia
[2]Novosibirsk State University, Novosibirsk, Russia
[3] These authors contributed equally: Tatyana A. Kurgina, Nina A. Moor
*Corresponding author O. I. Lavrik, Email: lavrik@niboch.nsc.ru ORCID: https://orcid.org/0000-0001-5980-8889

This PDF file includes:

Supplementary Figures 1 to 5

Supplementary Notes

Supplementary Note 1: **Analysis of the length of PARP1/PARP2-synthesized poly(ADP-ribose)**

Supplementary Note 2: **Testing of PARP1, PARP2-and HPF1 for DNA contamination.**

Supplementary references

Supplementary Figures

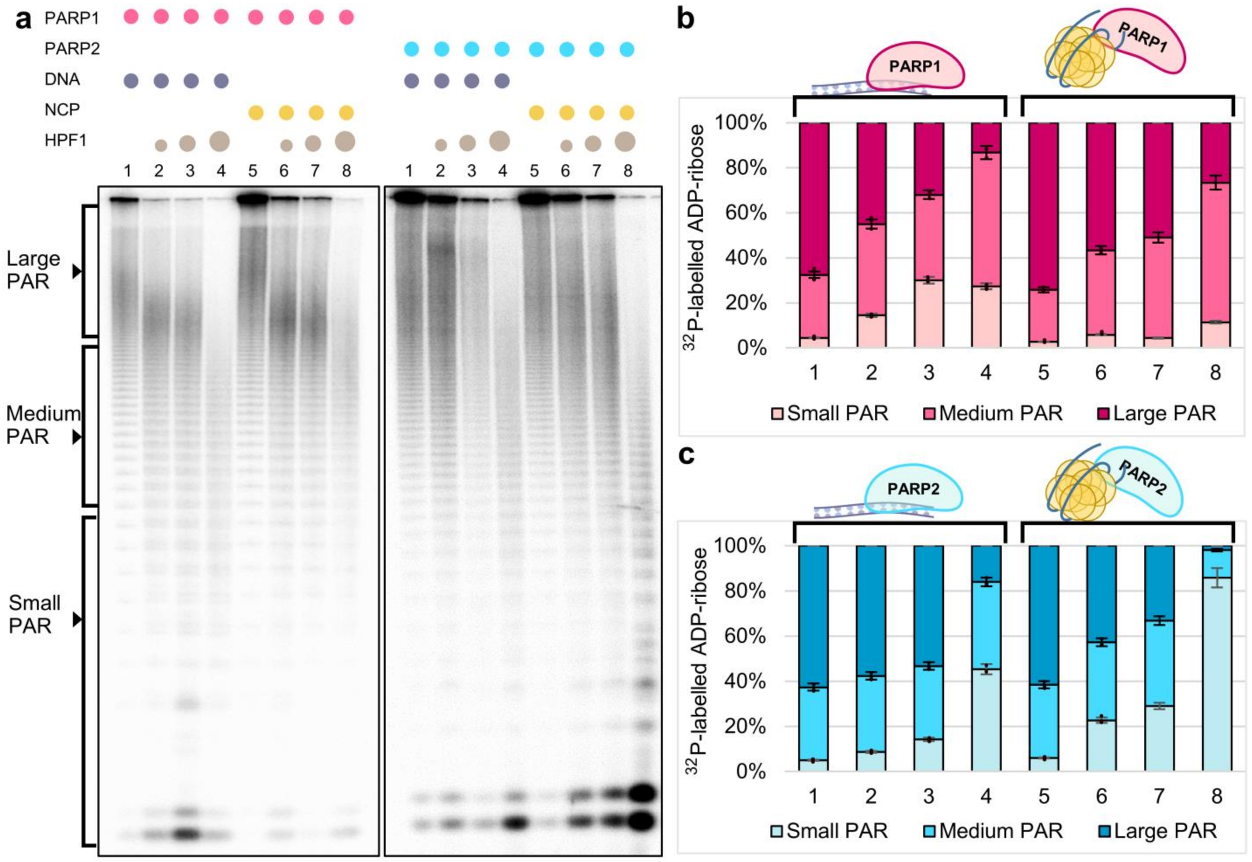

Supplementary Figure 1: PARPs 1 and 2 synthesize shorter PAR chains in the presence of HPF1.

a Autoradiograms of denaturing gels after separation of PAR chains synthetized by PARP1 (200 nM) or PARP2 (500 nM) (activated by 100 nM DNA or NCP) in the absence and presence of increasing concentrations of HPF1 (0.1, 0.5 or 2.5 µM) and detached from the modified proteins by proteolytic cleavage and alkaline hydrolysis (detailed in Supplementary Materials and methods). **b**, **c** Histograms show size distribution of PAR in the respective samples for PARP1 and PARP2 (the mean ± SD of three independent measurements). The amount of ^{32}P-radiolabel (%) in three polymer-size groups specified as small (1–10-mers), medium (1–30-mers) and large (more than 30-mers) is shown; high molecular weight bands near the loading wells were not considered.

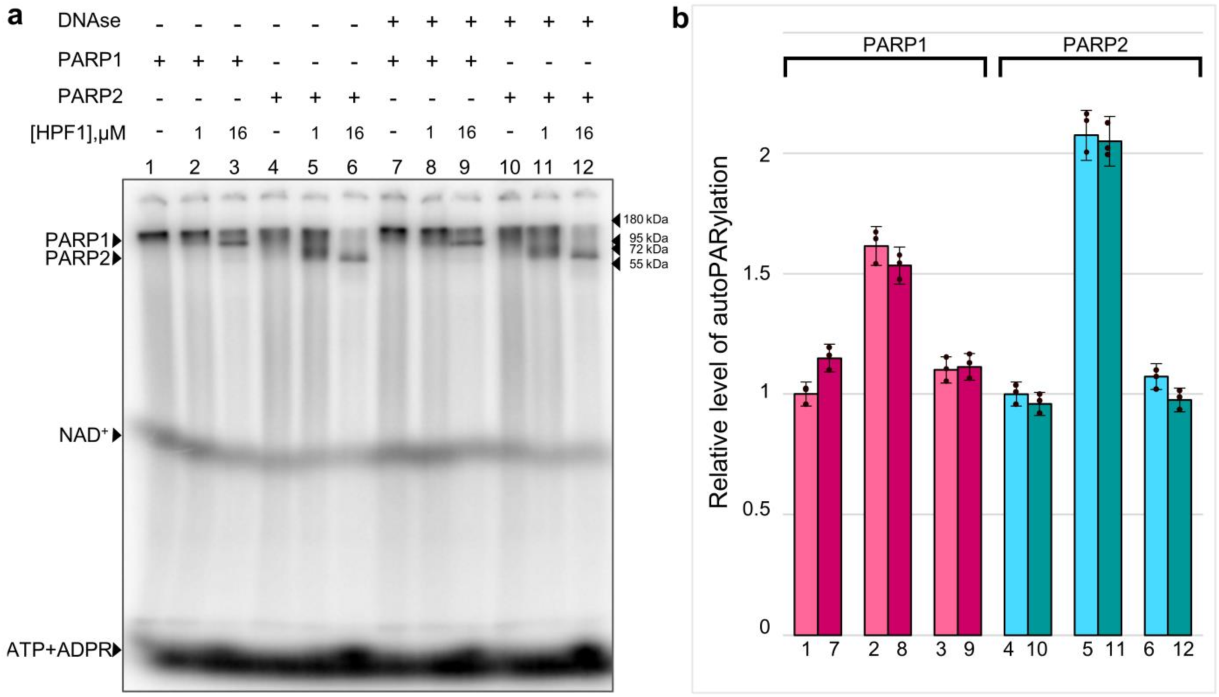

Supplementary Figure 2: Testing of PARPs and HPF1 for the absence of DNA contamination. a Covalent binding of ^{32}P-labelled ADP-ribose to proteins was performed by incubation of PARP1 and PARP2 (500 nM) with [^{32}P]NAD^{+} (1 μM), in the absence or presence of HPF1 (1 μM or 16 μM); samples 7-12 were preincubated for 30 min with 2 units of DNase I (Thermo Scientific, USA). The products were separated in 10% SDS-PAG. **b** Histograms present relative labelling levels (the mean ± SD of three independent measurements and individual data points) of PARP1 and PARP2 determined by normalization of the ADP-ribose amounts covalently bound to the given protein in the presence of HPF1, in the absence and presence of DNase (samples 2, 3, 7-9 for PARP1; samples 5, 6, 10-12 for PARP2) to the respective amount in the absence of HPF1 and DNase (sample 1 for PARP1 and sample 4 for PARP2).

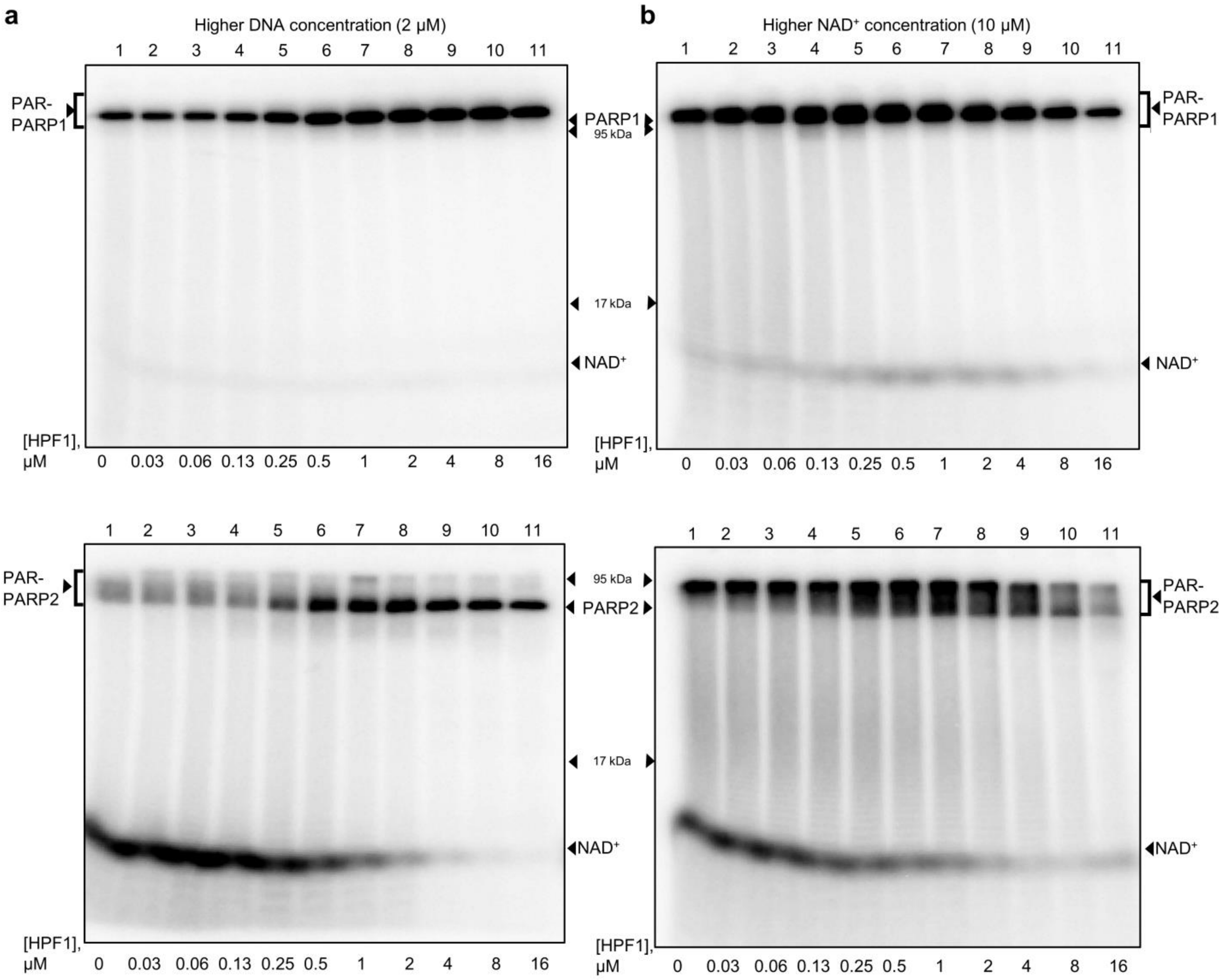

Supplementary Figure 3 (related to Figure 3): The stimulatory action of HPF1 on PARP1/PARP2 depends on the experimental conditions. Autoradiograms show covalent binding of [^{32}P]-labelled ADP-ribose to PARP1 and PARP2 after incubation of PARPs (500 nM) with 1 μM [^{32}P]NAD$^+$ and 2 μM DNA (**a**), or 10 μM [^{32}P]NAD$^+$ and 0.25 μM DNA (**b**), in the absence (sample 1) and presence of increasing HPF1 concentrations (from 30 nM to 16 μM, samples 2–10) and further separation of products in 20% SDS-PAG. The positions of PARylated protein and its native form are indicated on the left and right sides of the autoradiogram.

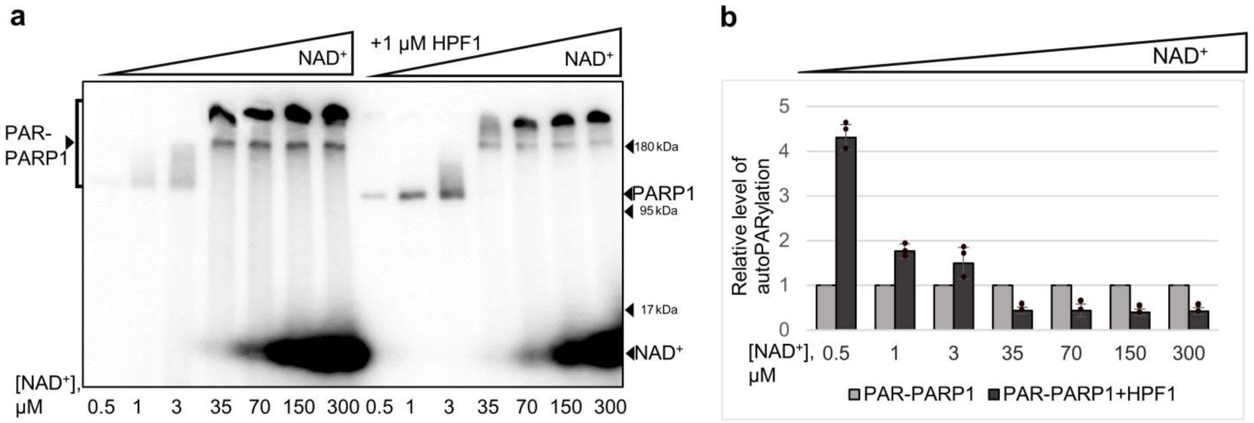

Supplementary Figure 4: The stimulatory action of HPF1 on PARP1 depends on the NAD$^+$ concentration. a Covalent binding of [^{32}P]-labelled ADP-ribose to proteins was performed by incubation of PARP1 (500 nM) with [^{32}P]NAD$^+$ (0.5–300 µM), in the presence of 31 bp DNA (250 nM) and HPF1 (1 µM); the products were separated in 10 % SDS-PAG. The positions of PARylated protein and its native form are indicated on the left and right sides of the autoradiogram. **b** Histograms present relative labelling levels (the mean ± SD of three independent measurements and individual data points) of PARP1 at defined NAD$^+$ concentrations determined by normalization of the ADP-ribose amounts covalently bound to PARP1 in the presence of HPF1 (samples 8–11) to the respective amount in the absence of HPF1 (samples 1-7).

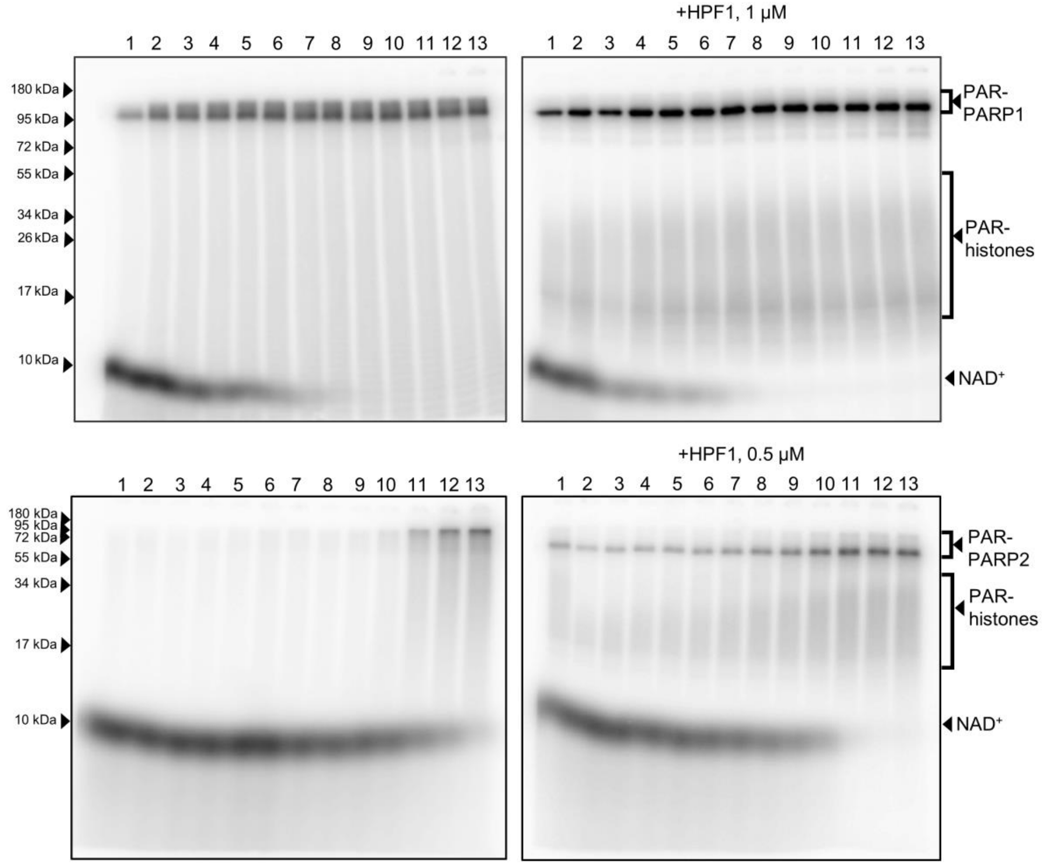

Supplementary Figure 5 (related to Figure 4): HPF1 modulates the auto- and heteromodification activities of PARP1 and PARP2 at the initial stage of the reactions. Autoradiograms show covalent attachment of [^{32}P]-labelled ADP-ribose to PARP1, PARP2 and histones upon incubation of PARPs (500 nM) with [^{32}P]NAD^{+} (10 µM) and 250 nM NCP, in the absence and presence of HPF1 (1 µM/0.5 µM in PARP1/PARP2-catalyzed reactions), and further separation of products in 20% SDS-PAG. The positions of PARylated proteins, their native forms and molecular weight markers are indicated.

Supplementary Notes

Supplementary Note 1 (related to Supplementary Figure 1): Analysis of the length of PARP1/PARP2-synthesized poly(ADP-ribose).

The length of poly(ADP-ribose) (PAR) synthesized by PARPs in the absence or presence of three different concentrations of HPF1 was analyzed. The PARylation reaction catalyzed by PARP1 (200 nM) or PARP2 (500 nM) was carried out in a 50 µl mixture, in the absence or presence of 0.1, 0.5 or 2.5 µM HPF1, with addition of 100 nM DNA or nucleosome for PARP activation. The reaction was initiated by addition of $[^{32}P]NAD^+$ to a final concentration of 300 µM. After incubation at 37°C for 45 min the reaction was stopped by addition of olaparib to 4 µM. The excess of $[^{32}P]NAD^+$ was removed by ultrafiltration and dialysis (against 50 mM Tris-HCl, pH 8.0, 150 mM NaCl), using a Viva-spin microconcentrator with 10 kDa membrane. The samples were subjected to alkaline hydrolysis by incubation with 40 mM NaOH at 37°C for 15 min, with subsequent addition of HCl to adjust pH to 7.5. Then, the reaction mixtures were treated with 20 µg of Proteinase K for additional 30 min incubation at 37°C. The bulk PAR was isolated by ethanol precipitation. The length of PAR chain was analyzed by electrophoresis in 20% denaturing PAG with subsequent phosphorimaging and quantification, using the Typhoon imaging system (GE Healthcare Life Sciences) and Quantity One Basic software (Bio-Rad). Note that the experiments were performed with a high NAD^+ concentration (300 µM), in order to diminish impact of decrease in the NAD^+ concentration due to its hydrolytic consumption enhanced by HPF1[1]. Results of autoradiographic analysis of bulk PAR synthesized in various conditions show that size distribution of PAR synthesized by both PARP1 and PARP2 was affected by HPF1 in a concentration-dependent manner: increasing the HPF1 concentration enlarged the relative amount of small PAR fraction.

Supplementary Note 2 (related to Supplementary Figure 2): Testing of PARP1, PARP2-and HPF1 for DNA contamination.

To exclude possible contamination of PARP1, PARP2 and HPF1 preparations with DNA, the PARylation reaction catalyzed by PARP1/PARP2 in the absence and presence of two different concentrations of HPF1 was explored with and without pretreatment of the reaction mixture with proteinase-free DNase. Relative levels of automodification determined for PARP1 and PARP2 in the DNase-treated and respective control samples were very similar, indicating that the activity detected in the absence of DNA/NCP is the basal (DNA-independent) activity.

Supplementary References

1. Rudolph, J., Roberts, G., Muthurajan, U. M. & Luger, K. HPF1 and nucleosomes mediate a dramatic switch in activity of PARP1 from polymerase to hydrolase. *Elife.* **10**, e65773 (2021).
