# Supplementary material for: Dual function of HPF1 in the modulation of PARP1 and PARP2 activities
Source: Commun Biol. 2021 Nov 3;4:1259. doi: 10.1038/s42003-021-02780-0 (PMC8566583; doi:10.1038/s42003-021-02780-0)
Supplement: Supplementary file 3 — Description of Additional Supplementary Files [file 42003_2021_2780_MOESM3_ESM.pdf]

Description of Additional Supplementary Files

File name: Supplementary Data 1.
Description: Source data and uncropped gels.
